# Vitamin D3 induces vitamin D receptor and HDAC11 binding to relieve the promoter of the tight junction proteins

**DOI:** 10.18632/oncotarget.17692

**Published:** 2017-05-08

**Authors:** Feng-Hua Liu, Shan-Shan Li, Xiao-Xi Li, Shuai Wang, Mao-Gang Li, Li Guan, Tian-Gang Luan, Zhi-Gang Liu, Zhan-Ju Liu, Ping-Chang Yang

**Affiliations:** ^1^ The Department of Gastroenterology, The Shanghai Tenth People's Hospital of Tongji University, Shanghai 200072, China; ^2^ The Research Center of Allergy & Immunology, Shenzhen University School of Medicine, Shenzhen 518060, China; ^3^ Longgang ENT Hospital, Shenzhen ENT Institute, Shenzhen 518116, China; ^4^ Affiliated Luohu Hospital, Shenzhen University, Shenzhen 518001, China

**Keywords:** intestine, epithelium, barrier function, vitamin D, histone deacetylase

## Abstract

Intestinal epithelial barrier dysfunction and vitamin D (VitD)-deficiency play a critical role in a large number of diseases. The histone deacetylases (HDAC) are associated with a large number of immune diseases. This study tests a hypothesis that the interaction between VitD and HDAC is associated with the regulation of epithelial barrier functions. In this study, human intestinal epithelial cell line, T84 cells, was cultured into monolayers to be used as a model to test the epithelial barrier functions. We observed that in a VitD-deficient environment, the T84 monolayer barrier function was compromised. Exposure to calcitriol (the active form of VitD3) in the culture increased the expression of VitD receptor (VDR) in T84 cells. In a VitD-sufficient environment, VDR formed a complex with histone deacetylase-11 (HDAC11); the complex was markedly decreased in a VitD-deficient environment. We also observed that significantly more binding of HDAC11 to the promoter of the tight junction proteins inhibit the gene transcription activities of these loci in the VitD-deficient environment, which were abolished by the presence of calcitriol in the culture. In conclusion, the interaction between VDR and HDAC11 plays a crucial role in the maintenance of the epithelial barrier integrity.

## INTRODUCTION

Intestinal epithelial cells connect each other by the tight junction (Tj) complexes. The epithelial cell bodies and the Tjs form the intestinal epithelial barrier. This barrier only allows water and some substances (usually no harm to the body) with small molecular weight to pass through [1]. It has been recognized that the epithelial barrier dysfunction is associated with a number of intestinal diseases, such as food allergy [2] and inflammatory bowel diseases [3]. The underlying mechanism has been investigated extensively, but is not fully understood yet.

In the disorders with intestinal epithelial barrier dysfunction, defect is found in the Tj, such as the decrease in the expression of Tj proteins, which results in the hyperpermeability to macromolecular substances [4]. Proteins with competent antigenicity may pass through the defect Tjs to arrive at the deep regions of the intestinal tissue, where the antigens may contact immune cells to initiate unwanted immune responses and induce immune inflammation [5]. Yet, how the pathogenic factors suppress the expression of Tj proteins is to be further investigated.

Lipopolysaccharide (LPS) is a component of the Gram-negative bacterial cell wall, which distributes extensively in the living environment. Published data indicate that LPS can impair the epithelial barrier function [6]. However, it is possible that healthy people also contact LPS because LPS also exists in the healthy intestinal tract. Why LPS does not impair the barrier functions in healthy condition is unclear. Whether LPS impair barrier function also via other pathways is to be further investigated.

Vitamin D (VitD) deficiency is associated with the pathogenesis of a number of diseases [7], such as allergic diseases [8], inflammatory bowel disease [9] and autoimmune disorders [10]. VitD is fat-soluble and can be absorbed easily by cells. The major function of VitD is to facilitate the absorption and metabolism of calcium and the bone health [11]. In the recent years, it has been revealed that VitD also has immune regulatory functions and contributes to the homeostasis in the body [12]. Published data also indicate that VitD is involved in the regulation of the epithelial barrier functions [13, 14]. Yet, the underlying mechanism remains to be further investigated.

Histone deacetylases (HDAC) play important roles in the regulation of gene expression. HDAC are a family of enzymes, including 11 subtypes in human beings. HDAC remove acetyl groups from an ε-N-acetyl lysine amino acid on a histone to allow the histones to wrap the DNA more tightly. It is reported that HDAC are associated with the pathogenesis of immune disorders [15] as well as the epithelial barrier dysfunction [16]. The interaction between VitD receptor (VDR) and HDAC is also involved in immune diseases [17]. Therefore, we hypothesize that the interaction between VitD and HDAC is associated with the regulation of epithelial barrier functions. To test the hypothesis, we performed this study. The results showed that VDR formed a complex with HDAC11 in epithelial cells. VitD deficiency resulted in less expression of VDR to render more HDAC11 to interfere with the expression of Tj proteins and thus compromised the epithelial barrier functions.

## RESULTS

### LPS-impaired T84 monolayer barrier function can be prevented by VitD

**Figure 1:**
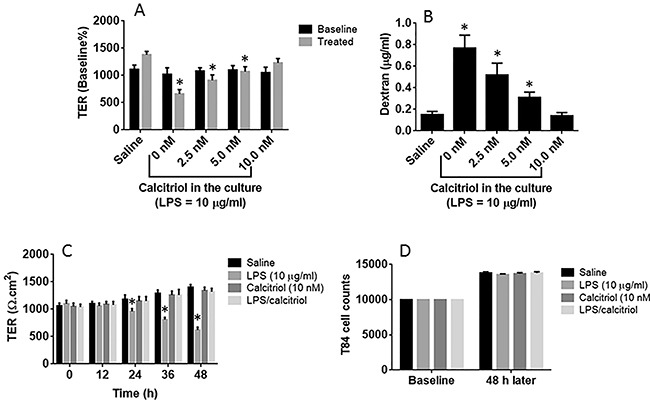
VitD blocks LPS-induced epithelial barrier dysfunction **(A-B)** the confluent T84 monolayers were stimulated with LPS (10 μg/ml) for 48 h with or without the presence of calcitriol at concentrations as denoted on the X axis. The bars indicate the TER levels **(A)** of T84 monolayers and the dextran levels in the basal chambers of Transwells **(B)**. **(C)** the bars indicate the TER levels of T84 monolayers recorded at the time points denoted on the X axis after the indicated treatment. **(D)** T84 cell counts after culture for 48 h with the indicated treatment. The data are presented as mean ± SD. *p<0.01. The data were summarized from 3 independent experiments.

Since VitD plays a role in the maintenance of the homeostasis in the body [18, 19], we firstly observed the role of VitD in the maintenance of the intestinal epithelial barrier function. Prompted by published data [20], we treated T84 cell monolayers with lipopolysaccharide (LPS), with or without the presence of calcitriol (the active form of VitD3). The results showed that LPS decreased the transepithelial electric resistance (TER) and increased the permeability to dextran in a dose- and time-dependent manner without the presence of calcitriol, indicating that LPS impaired the epithelial barrier function. The presence of calcitriol prevented the effects of LPS on impairing the T84 monolayer barrier function (Figure 1A-1C). To elucidate if the treatment affects the cell proliferation, we counted the cells by flow cytometry. The results showed no significant difference in the cell counts between groups at the end of experiments (Figure 1D).

### Expression of VDR is associated with the barrier function of T84 monolayers

**Figure 2:**
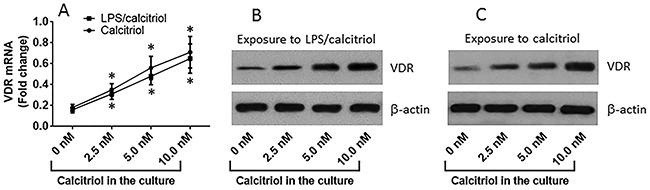
VitD up regulates VDR expression in T84 cells **(A)** the curves (mean ± SD, summarized from 3 independent experiments) indicate the VDR mRNA levels in T84 cells after exposure to calcitriol or LPS (10 μg/ml) and calcitriol in the culture for 2 days. **(B-C)** the immune blots indicate the protein levels of VDR in T84 cells after exposure to LPS and calcitriol **(B)** or exposure to calcitriol alone **(C)**. The data of B-C are from one experiment representing 3 independent experiments. *p<0.01, compared with the “0 nM” group.

**Figure 3:**
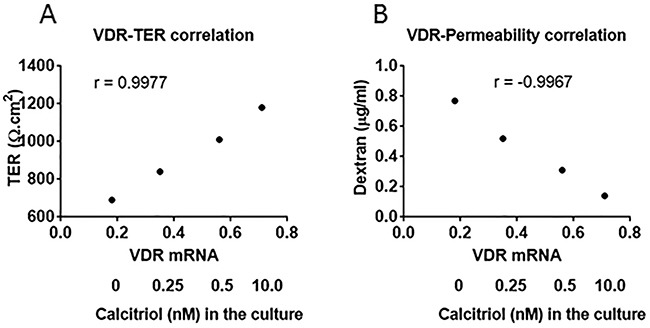
Correlation between VDR mRNA and T84 monolayer barrier function The dot plots show the correlation between VDR and TER, VDR and permeability of T84 monolayers. The T84 monolayers were exposed to LPS (10 μg/ml) and calcitriol at gradient concentrations for 48 h. The data are from one experiment representing 3 independent experiments.

To observe the effects of VitD on regulating the expression of VDR in T84 cells, we treated T84 cells with calcitriol or both calcitriol and LPS in the culture for 48 h. After assessing the barrier function, the cells were analyzed by RT-qPCR and Western blotting. The results showed that exposure to calcitriol markedly increased the expression of VDR in T84 cells in a dose-dependent manner (Figure 2A). Exposure to both calcitriol and LPS also increased the expression of VDR in T84 cells, which was similar to that exposure to calcitriol alone (Figure 2). A correction test was performed with the data of VDR expression and the barrier function of T84 monolayers. The results showed that a positive correlation was identified between VDR and TER of T84 monolayers (Figure 3A) and a negative correlation was identified between VDR and the permeability of T84 monolayers (Figure 3B). The results demonstrate that VDR plays a crucial role in the maintenance of the T84 monolayer barrier functions.

### Expression of VDR is correlated with the expression of Tj proteins

**Figure 4:**
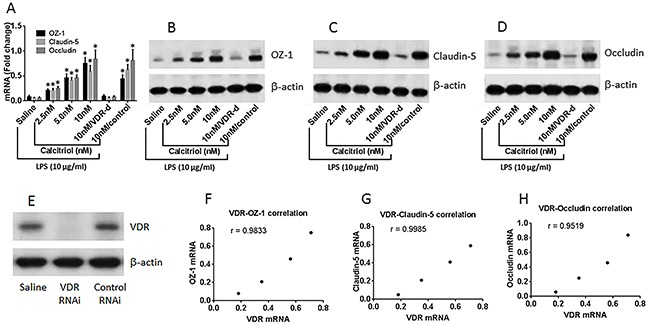
VitD reverses the LPS-suppressed Tj protein expression **(A-D)** the results of Tj protein expression in T84 monolayers [with or without the VDR gene knockdown (VDR-d)] after exposure to LPS (10 μg/ml) in the culture with or without the presence of calcitriol (denoted on the X axis). Control: T84 monolayers were treated with control shRNA. The data of bars (mean ± SD; *p<0.01, compared with the saline group) are summarized from 3 independent experiments. The immune blots are from one experiment representing 3 independent experiments. **(E)** RNAi results of VDR in T84 cells. **(F-H)** the dot plots show the correlation between VDR mRNA and the mRNAs of Tj proteins in T84 monolayers.

To look into the mechanism by which LPS impairs the T84 monolayer barrier function, the expression of Zonula occludens-1 (ZO-1), claudin-5 and occludin in T84 monolayer was assessed. The results showed that after exposure to LPS for 48 h, the expression of ZO-1, claudin-5 and occludin was markedly decreased, which was blocked by the presence of calcitriol (Figure 4A-4E). The expression of VDR was positively correlated with the expression of ZO-1, claudin-5 and occludin in T84 cells (Figure 4F-4H).

### VDR binds HDAC11 to prevent it from binding the promoters of ZO-1, claudin-5 and occludin

**Figure 5:**
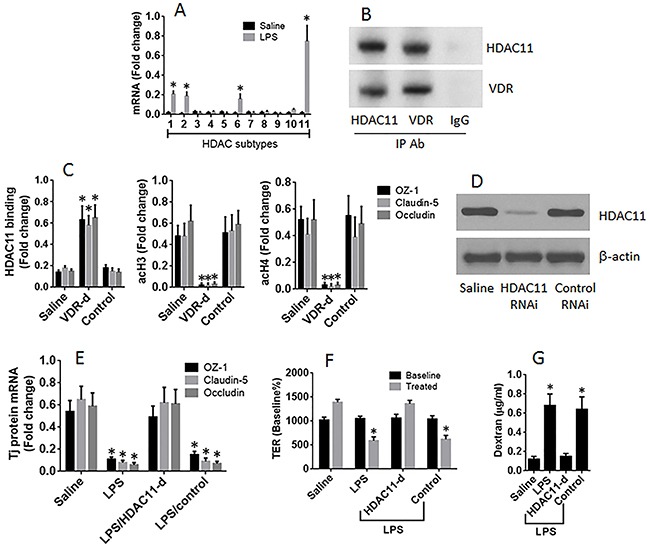
HDAC11 mediates the effects of LPS on suppression of T84 monolayer barrier function T84 monolayers (wild type, VDR-deficient or HDAC11-deficient) were cultured in the presence or absence of LPS (10 μg/ml) for 48 h. **(A)** the HDAC11 mRNA levels. **(B)** a complex of VDR and HDAC11. **(C)** the levels of HDAC11, acetylated histone (acH) 3 and acH4 at the promoter loci of Tj proteins. **(D)** the results of HDAC11 RNAi of T84 cells. **(E)** the mRNA levels of Tj protein in T84 cells. **(F-G)** the TER **(F)** and permeability to dextran **(G)** of T84 monolayers. The data (mean ± SD) were summarized from 3 independent experiments. *p<0.01, compared with the saline group.

The results reported above imply that the gene transcription of the Tj protein is interfered during the exposure to LPS in the culture. To test this, we screened the expression of HDAC in T84 cells after exposure to LPS. The results showed that LPS enhanced the expression of HDAC1, HDAC2, HDAC6 and HDAC11 in T84 cells, among which the expression of HDAC11 was uniquely higher (Figure 5A). Immunoprecipitation results showed a complex of VDR and HDAC11 in the T84 cells (Figure 5B). ChIP assay results showed that the levels of HDAC11 were significantly higher at the promoter loci of ZO-1, claudin-5 and occludin, respectively, in T84 monolayers exposed to LPS as compared with those treated with saline (Figure 5C). The results implicate that HDAC11 may be the factor to inhibit the gene expression of the Tj proteins in response to stimulation of LPS, which may be restricted by VDR. To test this, we performed ChIP assay. The results showed that knockdown of VDR significantly enhanced the levels of HDAC11, and down regulated the levels of acetylated histone (acH)3 and acH4, at the promoter loci of Tj proteins (Figure 5C). Secondly, we knocked down the gene of HDAC11 in T84 cells (Figure 5D). The HDAC11-deficient T84 monolayers were prepared and exposed to LPS in the culture for 48 h. The results showed that the exposure to LPS did not affect the expression of Tj proteins (Figure 5E) and did not impair the barrier functions of the HDAC11-deficient T84 monolayers (Figure 5F-5G), indicating that HDAC11 plays a crucial role in the mediation of the effects of LPS on impairing the expression of the Tj protein of T84 cells.

## DISCUSSION

Intestinal epithelial barrier dysfunction is associated with the pathogenesis of a number of intestinal diseases; to elucidate its causative factors is of significance. The present study revealed that the VitD deficiency played a role in the induction of T84 monolayer barrier dysfunctions. After culturing in a VitD deficient environment, the expression of VDR was decreased, resulting in the high binding rate of HDAC11 to the promoter loci of Tj proteins. The binding of HDAC11 resulted in repressing the gene transcription of Tj proteins in T84 cells and the dysfunction of the epithelial barrier. Such a dysfunction of epithelial barrier could be prevented by the presence of calcitriol, the active form of VitD 3.

In addition to contributing to the calcium absorption and metabolism, it has been found that VitD also contributes to the maintenance of the homeostasis in the body, although the underlying mechanism is not fully understood. For example, the VitD deficiency is correlated with the inflammatory condition in rheumatoid arthritis [21]. Administration with VitD supplements prevents the exacerbation of chronic obstructive pulmonary disease [22]. Our study adds mechanistic information to this study area that the deficiency of VitD can induce abnormality of the expression of VDR in T84 cells. T84 monolayers with an insufficient expression of VDR showed the epithelial barrier dysfunction in response to LPS.

LPS commonly exists in the intestinal tract; it does no harm to the healthy intestinal tissue. However, cumulative reports indicate that LPS can induce intestinal epithelial barrier dysfunction. Ling et al reported that LPS impaired Caco-2 monolayer barrier function [23]. Zhao et al found that the lung epithelial barrier dysfunction in rats with sepsis [24]. The possible mechanism is that LPS induces macrophages to release proinflammatory mediators, such as IL-8, IL-6 or tumor necrosis factor, to impair the epithelial barrier function [23, 25]. Yet, why LPS does not impair the healthy epithelial barrier has not been fully elucidated. Our data show that LPS does impair the barrier function, but only in a VitD deficient environment. The LPS-induced epithelial barrier dysfunction can be antagonized by the presence of VitD.

The data show that VitD deficiency is associated with the decrease in Tj protein expression in T84 monolayers. Tj proteins are the major components of the epithelial barrier. Published data have shown a number of factors that are associated with the Tj abnormality-associated epithelial barrier dysfunction. Exposure to proinflammatory cytokine, such as IL-1β, can inhibit the expression of claudin-3 in intestinal epithelial cells [26]. Our data have added novel evidence to this point by showing that VitD deficiency is one of the causative factors of epithelial barrier dysfunction. Others also found that VDR-deficient mice showed high epithelial barrier permeability [13]. Do et al reported that VitD protected the epithelial barrier function via inhibiting the expression of the long isoform of myosin light chain kinase, in which VitD disrupts the nuclear factor (NF) -κB p65 binding to 3 κB sites in long MLCK gene promoter [27]. Kong et al found that VitD could promote epithelial cell migration, suggesting that VDR plays a role in the healing capacity of the colonic epithelium [14]. Not only playing a role in maintaining the barrier function, Liu and Wu observed that the VDR on intestinal epithelial cells were critical in attenuating experimental colitis as well [28, 29]. Our data further demonstrate the mechanism by which VDR forms complexes with HDAC11 to attenuate the HDAC11 binding to the promoters of Tj proteins in epithelial cells, and thus antagonize the effects of HDAC11 on interfering with the barrier function.

Since the epithelial barrier dysfunction is associated with the pathogenesis of many diseases, to improve the barrier function is expected to facilitate the disease treatment. Our data indicate that the VitD deficiency affects epithelial barrier function by repressing the Tj protein expression, which can be improved by the presence of VitD at sufficient concentrations. The results suggest that in screening the pathogenic factors for diseases associated with epithelial barrier dysfunction needs to take the VitD deficiency into account. If the VitD deficiency is accompanied with diseases with epithelial barrier dysfunction, to adjust the VitD to physiological levels is expected to facilitate the therapeutic effects.

## MATERIALS AND METHODS

### Reagents

The LPS, FITC-labeled dextran, calcitriol and ChIP kit were purchased from Sigma Aldrich (St. Louis., MO). The antibodies of VDR, ZO-1, claudin-5, occludin, HDAC11, acH3, acH4, shRNA kits of VDR and HDAC11 were purchased from Santa Cruz Biotech (Santa Cruz, CA). The reagents for Western blotting and RT-qPCR were purchased from Invitrogen (Carlsbad, CA). The reagents used in this study contained <0.2U endotoxin/10 μg reagents as assessed using the Limulus assay (Limulus amebocyte lysate QCL 1000, Bio Whittaker, Walkersville, MD, USA).

### Preparation of T84 cell monolayers

T84 cells (a human intestinal epithelial cell line, passage 38 to 42) were purchased from ATCC (Manassas, VA) and cultured in Dulbecco's modified Eagle medium supplemented with 10% fetal bovine serum, 100 U/ml penicillin, 0.1 mg/ml streptomycin and 2 mM L-glutamine at 37 °C and 5% CO_2_ environment. The cells were seeded on the filter of inserts of Transwells. The transepithelial electric resistance (TER) was recorded with an Ohmmeter. When the TER reached 1000 Ω.cm^2^, the monolayers were regarded confluent and used for further experiments.

### T84 cell proliferation assay

The T84 cells were seeded at a density of 10,000 cells/well in 24-well plates. The cells were cultured in the presence of saline, or LPS, or calcitriol, or LPS and calcitriol, for 48 h. The cells were collected at the end of culture. The cells were counted with a flow cytometer (FACSCanto II, BD Bioscience).

### Assessment of the permeability of T84 monolayers

FITC-labeled dextran (MW=40 kDa) was added to the upper chambers of Transwells with confluent T84 cell monolayers at 20 μg/ml. Samples were taken from the basal chambers 48 h later. The contents of the dextran in the samples were determined with a fluorescent spectrometer and presented as dextran/ml.

### Assessment of the effects of LPS on regulating T84 monolayer barrier function

LPS was added to the upper chambers of Transwells with confluent T84 monolayers at gradient concentrations. The TER and permeability to dextran of the T84 monolayers were assessed 48 h later as described above.

### Real time quantitative RT-PCR (RT-qPCR)

T84 cells were collected at the end of the experiments and subjected to RNA extraction. The cDNA was synthesized with the RNA and a reverse transcription reagent kit. qPCR was performed in a qPCR device (MiniOpticon, Bio-Rad Life Science) with the SYBR Green Master Mix and primers as listed in Table 1. The results were calculated with the 2^-DDCt^ method and normalized to fold change against control groups.

**Table 1:** Primers used in the present study

Molecules	Forward	Reverse
VDR	gccatccacaattccaggtc	tcccacccgatatcaccttg
ZO-1	ccagcatcatcaacctctgc	catgcgacgacaatgatggt
Claudin-5	gctgtttccataggcagagc	ccctgccgatggagtaaaga
Occludin	tgtagggaggagggaaagga	tggtccaatcacagctcact
HDAC 1	cttccccaacccctcagatt	atccctttcacccagacctg
HDAC 2	tggtgtccagatgcaagcta	gccacatttcttcgacctcc
HDAC 3	acttcgagtactttgcccca	ggcacgtcatgaatctggac
HDAC 4	tgggaaacgagcttgatcct	catctggtctcttttcggcg
HDAC 5	cagaagttgaacgtgggcaa	gtcctccaccaacctcttca
HDAC 6	tgtgctcccaatcctgacat	acgtactcagcactgtgaca
HDAC 7	caggcggaaggatggaaatg	atgcgttgctgtgaaaccat
HDAC 8	acgtgtctgatgttggccta	tcccagctgtaagaccactg
HDAC 9	caggcggaaggatggaaatg	atgcgttgctgtgaaaccat
HDAC 10	ggcctttgagtttgaccctg	cagcgtctgtactgtcatgc
HDAC 11	gtcttgcctgttcagtgcaa	tgcatccctgatttccacct
ZO-1 promoter-693 to -539	tggagggacagcattggaat	ccacaccccacattagacct
Claudin-5 promoter-833 to -584	caacatagtggggtcagggt	tgtcttcatgcgtctgtcct
Occludin promoter-1366 to -1177	cctggggtggtgatgtgtaa	tgctccaacgaaagactcct

### Western blotting

Total proteins were extracted from the T84 cells and quantitated by the BCA method. The proteins were fractioned by SDS-PAGE and transferred onto a PVDF membrane. After blocking with 5% skim milk for 30 min, the membrane was incubated with the primary antibodies of interest or isotope IgG (control) overnight at 4 °C, followed by incubating with the second antibodies (conjugating with peroxidase) for 1 h. The membrane was washed with Tris-buffered saline-Tween 20 three times after the incubation. The membrane was then treated with enhanced chemiluminescence. The results were photographed with an imaging device (UVI, Cambridge, UK).

### Immunoprecipitation (IP)

T84 cells were collected from related experiments and lysed with a lysing buffer. The samples were precleared by incubating with protein G agarose for 2 h at 4 °C. The samples were centrifuged to remove the agarose. The supernatant was incubated with antibodies of interest or isotype IgG overnight at 4 °C to precipitate the immune complexes. The immune complexes were collected by centrifugation and treated with eluting buffer. The proteins were subjected to Western blotting as described above.

### Chromatin IP (ChIP)

T84 cells were collected from related experiments and fixed with 1% formalin for 15 min. The cells were sonicated to shear the DNA to small pieces (200-500 bp). The samples were subjected to IP procedures as described above. The DNA in the samples was extracted with phenol/chloroform and precipitated by ethanol, and then analyzed by qPCR. The primers used in this experiment are listed in Table 1 (designed by the Sangong Biotech (Shanghai, China). The results are presented as fold change against the input.

### RNA interference (RNAi)

The genes of VDR or HDAC11 in T84 cells were knocked down by RNAi with reagent kits purchased from Santa Cruz Biotech following the manufacturer's instructions. The effects of RNAi were checked by Western blotting.

### Statistics

The data are presented as mean ± SD. The difference between groups were determined by Student t test or ANOVA followed by the Bonferroni correction if more than two groups. P<0.05 was set as a significant criterion.
